# Syphilinum or Luesinum, with Comments*Read before the Kansas State Homœopathic Medical Society, May, 1895.

**Published:** 1895-08

**Authors:** J. H. Allen

**Affiliations:** Logansport, Ind.


### SYPHILINUM OR LUESINUM, WITH COMMENTS.*

J. H. Allen, M. D., Logansport, Ind.

*Mind.*—One of the more characteristic mental symptoms of this remedy is loss of memory, and what is peculiar about it is that the patient generally remembers everything previous to his illness, while he forgets everything since his illness. He has much difficulty in concentrating his thoughts on particular subjects, yet can recollect consecutive events and details which have occurred many years previously to his sickness. He can remember proper names, such as persons, books, places, but cannot remember recent events (memory for single words improved, Lyssin). Cannot remember a previous line read, and has to ask the name of his most intimate friend (Medorrh.). He is despondent and doubts recovery; all his mental symptoms are aggravated at night, and he dreads the approach of night, especially on account of mental and physical exhaustion, has a feeling of going insane, or that he is going to be paralyzed, or he is apathetic, indifferent to the future; has a far-away feeling, as if the mind was scattered; dull and stupid and slow to comprehend, thus slow in speaking, finds answers slowly; weeps without a cause, is cross, irritable, peevish; mental symptoms are worse after sleep (similar to Lach.).

*Head.*—The headaches are peculiar; they are often called linear headaches, as they extend from one eye backward, or from the temple laterally, or from temple to temple, or they extend from some part of the head to the eye or to the ear; are worse by the heat of the sun; headaches in the side of the head with pulsation of the arteries; pains are intolerable, are often worse after sleep. Worse at noon, or rise to their climax at noon and gradually grow better toward night. Prostration after headaches and debility. We have another form of headache, commencing at four P. M., and at its worst from ten to eleven and ceasing at daylight. Headaches are often preceded by nervous

---

* Read before the Kansas State Homœopathic Medical Society, May, 1895.

chills and with bursting sensation in the vertex, as from a severe cold, or a sensation of weight on the occiput drawing the head back as if it were pulled back. Linear headaches, commencing in the forehead and extending in parallel lines backward, often forerunners of epileptic attacks, or we have a heavy cutting pain across the base of the cerebellum, or a dull feeling at the base of the brain; the attacks of headache come on periodically, especially in secondary syphilis, are accompanied with restlessness, sleeplessness, and nervousness, and are worse after excitement.

*Eye.*—Red papules around the left inner canthus, circumscribed areola, sensation of heat with pain in the lids or a pulsating pain in the orb of the eye; the eye pains are aggravated by lying on the left side (Lac-felinum, aggravated by lying on painful side), pain over right eye, often lasting for days and accompanied with drowsiness and lachrymation; the pain is worse by noise and by light; better by pressure and tight bandages; also worse at noon, and better when the sun goes down; adhesion of the lids; ptosis, paralysis of the lids where the eyes have a sleepy look from the drooping of the lids; paralysis of the muscles of the eye; chronic phlyctenular inflammation of the cornea, often accompanied with photophobia and profuse lachrymation, redness of the eye in delicate scrofulous children who have traces of hereditary syphilis (Graph., Proto-iod-merc.), intense itching of the canthus in ophthalmia neonatorum (Argent.). Eyes intensely swollen, with pus oozing from them; the eye pains are usually all worse at night and worse in the after part of the night, and are generally intermittent. The conjunctiva swollen, puffed, with a deep red color, often accompanied with chemosis and with immobility of the pupils; the tears are acrid, hot, scalding the cheeks; conjunctiva often looks red, injected, the lids everted, and the dread of light so great that he constantly wears a shade. In inflammation of the lids the patient often experiences a sensation of cold air blowing in them, or a horizontal line across the pupil, hindering sight, inflammation of the eyes; is better by cold applications.

*Tongue.*—The tongue is thickly coated; edges are indented or serrated by the teeth. One thing peculiar about the tongue is that it has two deep cracks or fissures running lengthwise on either side of the median line in secondary syphilis. Frequently we have one deep crevice running lengthwise following Mercury poison. You will often have to differentiate between history of previous treatment by Mercury and the family history of hereditary syphilis. We have partial paralysis of the tongue so they cannot turn the food with the tongue properly, or it turns to one side when he attempts to protrude it; speech is heavy and sluggish; breath fetid, foul, stale, or moldy (Mer.). The tongue is often covered with herpetic eruptions or with foul bleeding ulcers, with lardaceous bottoms and bright fiery edges, as if cut down with a knife. These ulcers smart and burn like fire and often prevent sleep; saliva is profuse, stringy, viscid, often pouring from the mouth (like Calomel), has a sweetish taste, putrid, sickening odor which fills the whole house, very similar to salivation by Calomel; mouth symptoms are all worse at night; most of the eruptions in the mouth, throat, pharynx, and nasal passages are of a herpetic nature; liquids are more difficult to swallow than solids.

*Throat.*—It is a remedy that will be frequently needed in chronic hypertrophy of the tonsils in syphilitic patients. These patients are constantly having sore throats, especially from any sudden change of the weather or exposure to cold. They have chronic congested spots in the throat, adhesion of the soft and hard palate, cleft palate, ulcers of nares, roof of the mouth, or of the vomer bone; in chronic coryza the discharge is thin and watery; in catarrhal troubles the discharge is green and frequently excoriating, producing redness of the part passed over; abscess forming in the tonsils discharges profuse yellowish pus; large dry crusts are expectorated from the throat in the morning, or blocks of dry mucus from the nares, the parts often bleeding after their removal; the nasal symptoms are very similar to Aurum, and many cases have been cured by this remedy alone, where Aurum appears to be indicated. Aurum follows this remedy in nasal troubles, and it is frequently followed by Teucrum in otitus media.

*Face.*—Frequently we have spasmodic twitchings of many of the muscles, facial paralysis in the right side with jactitation. Face is pale, bloodless, often has a drawn appearance, hypocratic, pinched, drawn appearance. We have frequent neuralgia of the face, especially in the bones, very similar to Spigelia, or Kali-bichro., Lac-fel., or Stannum, or complete hemiplegia following severe attacks of neuralgia of the face.

*Teeth.*—The incisors serrated. Children's teeth we find cupped. They decay early at the edges of the gum, often breaking off, occasionally decaying as soon as they come through (like Kreosotum).

*Voice and Larynx.*—Almost complete aphonia. Chronic asthma coming on in summer, especially when the weather is warm during the hottest days of summer. Generally begins in the evening and passes off toward daylight. Patient often suffers very much soreness of the chest, with inability to remain in a recumbent position. Frequently these patients have a severe cough, which leaves them in the summer and is followed by asthma during warm weather (Chancroid, Phytolacca), and especially after rain or damp weather. Oppression of the chest is a marked symptom of the asthma, often coming with the sensation as if the sternum was drawn toward the back. The most severe attacks usually come in the after-part of the night, beginning usually about one o'clock and lasting until four. The wheezing and rattling are very severe, especially in syphilitic children (like Tart-emet.). The cough of this remedy is hard, dry, continuous, preventing sleep. It is always worse at night, and there is frequently a rasping or scraping sensation in the throat. Expectoration is white or yellowish, tasteless. When pus is expectorated, it is usually greenish or of a greenish-yellow. Often the asthma and aphonia precede the menstrual flow. Cough is made worse by lying upon right side (Medorrhinum), if accompanied with asthma, which is worse at mid-day.

*Spine.*—Spine sensitive the whole length of it to pressure. Caries of the spine, especially of the cervical portion, often with extreme curvature, usually laterally. Pain in the curvature, which is worse at night. Heaviness and dragging feeling, with dull pain in lumbar region of the spine. Cracks of the bone of the spine exuding bloody, offensive pus. Thickening of the spinal processes, sensitive to pressure. Pains are worse at night, beginning in the evening, terminating only at daylight; worse by motion and pressure, and better by heat. Psoas abscesses which discharge profuse offensive greenish pus. Enlargement of the cervical glands, tendency of abscesses in children between the ages of two and ten. Tendency to abscesses in pale, æenemic, bloodless, poorly-developed children.

*Pains.*—The pains are all severe, often excruciating and tormenting, preventing sleep, usually worse at night, except the headache. Rheumatic pains are sharp, burning often like fire, frequently awakening out of sleep, gradually increasing in severity, then decreasing, usually worse in the left side of the body.

*Extremities.*—Rheumatism of the deltoid muscle or the upper third of the humerus. "Run-around" on the fingers or thumbs, recurring annually. Often the part is red, swollen, sensitive, and burning. Swelling of the lower limbs, from the knees down, during the day, worse on standing, disappearing during the night. Severe pains in the long bones of the body, especially lower extremities and in the joints, pains are worse at night, worse by heat of bed, with redness, rawness and severe itching between toes (like Sil.). Rheumatism suddenly leaving the limbs and going to the heart, worse in the summer; usually or often accompanied with the deltoid form, impossible to raise arm at right angle. Aching in the limbs like growing païns in children. Sometimes the pains are better by heat of stove, but worse by warmth of bed, and worse from sundown to sunrise, often the part affected becomes numb. Ulcers on the shin-bones healed with salves or ointments left depressed scars, metastasis to the lungs soon after with râles and bronchial breathing, prolonged respiration and tight, dry cough without expectoration. Indolent ulcers of the lower extremities in emaciated and poorly-nourished subjects who have syphilitic taint, not relieved by any remedy; promptly cured by Syphilinum, one-thousandth potency. In old chronic forms of syphilis, the ulcers have a well-defined outline, and appear as if cut out with a chisel, generally very little discharge and the pus is greenish or watery, and the bottom of the ulcer appears granular, the tissue around them is usually dark red, discolored, and irritated. Seldom do they itch and are not very sensitive. False granulations in chronic forms prevent healing; they bleed slightly when touched, and if healed by local means leave deep scars that look as if they had been burnt. They appear on shin-bones. Periostoma of lower extremities. Toenails have a tendency to grow distorted and ridged or furrowed, or to grow under and are painful and sore at the change of moon, especially full moon. The nails are as thin as paper, break and split easily, or the edges are imperfect, notched, and in these notches is a film of thin material like tissue paper. The finger tips are club-shaped and the knuckles large, the lines in the palmar surface deep and furrowed, ulcers appear in mouth and on tongue at or about the menstrual period that are sensitive and painful, very similar to Nitric-acid and worse at night and at change of moon.

*Fever.*—Chills accompanied by pain in the head, face has a blue appearance, whole body cold, desire to be covered with blankets yet could not get warm, sleepy during the chill. Nervous chills preceded by aching pains in the head or limbs, with a feeling of heaviness in the limbs, cross, irritable, and peevish, pain begins at four P. M. and growing worse until midnight, then growing better.

*Hot Stage.*—Hot fever, dry, parched lips, and great thirst. During fever intensely hot, wants to throw off covers and get his feet out of bed. Intensely hot feeling and great debility, perspires often during the fever, or the fever is followed by profuse debility, night-sweats, patient waking up almost exhausted.

*Sexual Organs, Male.*—Chancre size of a split pea on corona glandis, edges raised and bottom covered with lardaceous deposit, mucous membrane purplish, aching of genitals, bubo swollen, purple, more painful at night and usually accompanied with night-sweats. Phagedænic forms of chancre.

*Female Sexual Organs.*—Ulcers on labia painful, similar to Nitric-acid, though not so sensitive and do not bleed so easily. The leucorrhœa is usually profuse, thin, and watery, soaking through napkin or fully as bad as Alumina, if not worse; flowing worse at night and generally acrid and excoriating and producing itching even to pruritis of a severe form. Often the leucorrhœa is accompanied with severe pain in right ovary, though either ovary may be effected, and the most characteristic pain is a sharp, knife-like pain; the breasts are usually sore, tender, and somewhat enlarged during menses, similar to Puls. or Conium.

*Cough.*—Dry, hacking cough coming on in the spring of the year. Very regular and accompanied with nose-bleed or hemorrhage from the uterus. The cough is irritable, dry, hacking, worse at night or it wakes her at a regular hour after midnight, paroxysmal, like whooping-cough without the whoop. No part of the wind-pipe can be touched without exciting cough (Lachesis), phlegm has to be swallowed, impossible to expectorate it, similar to the Kalis. It is better lying on the right side, worse lying on the left side, like Phosphorus. It has also an aggravation at three A. M. The patient usually dreads the night on account of the cough, as the cough exhausts her so. A typical syphilitic patient is usually, though not always, light complected, fair skin, and more or less freckles, blue or brown eyes, light hair. They are usually thin and of the ænemic order, having poor vitality. They are easily exhausted by exertion, they have a lack of reactive force in recuperating from disease or responding to the actions of foods, medicines, or anything. They suffer either from a complete loss of assimilation or the lack of it in some way. Children appear healthy when they are born, are fat and chubby, but frequently die before they are a year old from marasmus, especially where psora is combined with latent syphilis. I have frequently called a halt in diseases of children that must have proved fatal, by giving a few doses of Swan's potencies of Syphilinum. This I find more especially in marasmus and summer complaints of children. In tuberculosis the children, where it would seem to be well indicated, receive no marked benefit. One reason that I have in mind that I might mention here is that tuberculosis is not so much based upon syphilis as we suppose. It has, I think, more of a basis of psora with chancroid, especially where the chancroid has been suppressed. It is, you know, very easily suppressed, so much so, that the dominant school considers it a very trifling affair, while they look upon the hard chancre as something terrible. The truth of the matter is chancroid is the miasm most to be dreaded, as it has such an affinity for psora, and the combination is, in my estimation, the basis of cancer, tuberculosis, wasting diseases, Bright's disease, severe neuralgias, much of our insanity, and very stubborn forms of skin troubles. I dread chancroid, as the incurable cases, I find, and the ones I cannot get at, usually have this miasm as a basis, especially in patients where it has been suppressed. When these patients fall they usually go down with a crash never to rise again, and a metastasis means a malignancy which is labelled with the stamp of death. Syphilis does not readily combine with psora, but is a miasm of a very aristocratic nature, preferring to live alone, weds not, and when he is present he usually heads the list. Much has been said, *pro* and *con*, concerning the possibility, or rather probability, of this remedy being indicated (or any nosode for that matter) without the miasm being present in the system. I am inclined to answer with the minority, and that is they are never indicated unless the miasm be present, either in the acute or chronic form. The presence of a miasm in the system develops a dyscrasia, is usually followed by idiosyncrasy, and the latter peculiarity in patients is a very interesting study as to the relations they bear to nosodes. We often have to cure the predisposition to disease by the use of a nosode, by striking at the fountain head. Is this not truly wonderful, death lurks in the very means of grace and *vice versa?*

I cannot too highly praise the great benefit to be derived from the use of nosodes if they are properly administered, but if placed in the hands of incompetent men there is great danger of doing more harm than good. He who is a student of *The*

*Organon* and a true follower of Hahnemann and the law of *similia* can safely use them, as he knows well the difference between a true picture of *similia* and a pathological relationship. The law of *similia*, if applied to sick human beings, must either cure or palliate in a way known only to homœopathic physicians. It is only that which is out of law that kills, or that which is not (or cannot be), through our ignorance, brought into true relationship with law. The great law of *similia* draws no lines of demarcation. There is no class of remedies that it can bar out, if man is an epitome of this universe then any force in the universe may derange or disturb him, and it may require one or more of any of the forces to restore him to a normal condition again, and the laws that make men sick act as beautiful as those that make men well. The pendulum of the third great law of motion sways as far to the left as it does to the right, the force of the poppy juice makes men numb and dead to pain, but when reaction comes it makes them doubly alive to it. So in our study of the action of any of the nosodes we must remember that they have the qualities of all forces and the characteristics of all remedial agents—that is, they must be disease-producers before they can be disease-curers.

My article is already too long to go into detail as to when nosodes should be used, and when they should not be used, simply to say to you in conclusion study well the miasm syphilis and the combination with other miasms, especially the relation it bears to psora, and you will find in it a remedy that will be more frequently the simillimum than you have heretofore thought. I have heard many an objection to the use of nosodes. One in particular, that they are filthy and unclean is, I think, a very lame excuse, and recalls to memory the vision of Apostle Peter on the different kinds of meats that were jumbled together in the great sheet that was let down from heaven, a wonderful lesson to Apostle Peter, and let us all take a lesson from the fact that nothing is unclean to the man of science and especially to the law of *similia*. The law that leaves dead and inanimate matter behind by the power of potentiation, and brings forward the forces that made it at its creation, is the law that must govern us. Man's judgment is blind judgment because he judges by and through his material senses which brings him only into relationship with material things. Law judges like a god, for it is moved by an omnipotent hand. There is nothing unclean in the fifty-thousandth potency of anything, and you cannot afford to use a nosode made lower. Then let us be forever silent on the question of uncleanness in nosodes, and be men of science, pushing forward and upward and onward, led by the mighty and unerring hand of law, and lift our science to a higher standard and float over its citadel the banner of *similia.*

